# Identification and chemical structure elucidation of synthetic cannabinoids samples seized in Kuwait during 2019–2023 using GC–MS and NMR spectroscopy

**DOI:** 10.1093/fsr/owae026

**Published:** 2025-04-23

**Authors:** Abdullah Al-Matrouk, Khaled Y Orabi

**Affiliations:** Narcotic and Psychotropic Laboratory, Department of Criminal Evidence, Ministry of Interior, Kuwait City, Kuwait; Department of Pharmaceutical Chemistry, College of Pharmacy, Health Sciences Center, Kuwait University, Safat, Kuwait

**Keywords:** synthetic cannabinoids, Kuwait, nuclear magnetic resonance

## Abstract

Cannabinoids, a class of chemical compounds, interact with cannabinoid receptors and are categorized into endocannabinoids, phytocannabinoids, and synthetic cannabinoids (SCs) based on their origin. Among these, SCs constitute the largest and most structurally diverse group of novel psychoactive substances (NPS), with around 280 compounds identified globally. They exhibit a high binding affinity to cannabinoid receptors CB1 and CB2, which are distributed throughout the central nervous and immune systems, leading to more potent psychoactive and toxic effects compared with their natural counterparts. Various adverse effects associated with SCs include hypothermia, analgesia, catalepsy, psychosis, respiratory depression, cardiac arrest, nephrotoxicity, acute cerebral ischemia, seizures, and mortality. In a previous study, we reported the detection of several NPS in Kuwait using gas chromatography–mass spectrometry and liquid chromatography-tandem mass spectrometry techniques. However, the identification was tentative, highlighting a limitation of these methods. To address this, the current study aimed to fully identify 17 seized SC samples. Thin-layer chromatography was initially employed to assess the purity of the samples. Twelve pure samples (AKM-1–AKM-12) underwent nuclear magnetic resonance analyses, including ^1^H, ^13^C, DEPT 45°, 90°, 135°, COSY, HSQC, and HMBC experiments. The identities of five samples (AKM-1, 5, 6, 8, 10) were confirmed as MDMB-4en-PINACA, one sample (AKM-2) as 4F-MDMB-BUTICA, one sample (AKM-3) as MPHP-2201, and three samples (AKM-4, 9, 11) as MMB-022. Additionally, two samples (AKM-7, 12) were identified as FUB-144. This comprehensive approach enhances the accuracy of SCs identification compared with previous studies, emphasizing the importance of employing nuclear magnetic resonance alongside other spectral methods for a more robust analysis.

## Introduction

Cannabinoids are chemical compounds that act on the cannabinoid receptors and are classified according to their source of production into endocannabinoids, phytocannabinoids, and synthetic cannabinoids (SCs) [1, 2]. SCs are the largest growing and most structurally diverse class of novel psychoactive substances (NPS) with approximately 280 compounds identified worldwide, as reported by the United Nations Office of Drugs and Crimes (UNODC) [3–5]. Mainly, they are full receptor agonists with a high binding affinity to the cannabinoid receptors CB1 and CB2, which are distributed throughout the central nervous system and immune system, respectively. As a consequence, this will result in producing psychoactive effects that are similar to or, in many cases, even more potent than that of the mainstream drugs they are mimicking (i.e., 1–100× higher affinity than tetrahydrocannabinol (THC)) [6–8]. Such pharmacological actions of SCs can result in severe physical and psychological effects, when compared with abusers of natural cannabis. Some of these toxic effects include hypothermia, analgesia, catalepsy, psychosis, respiratory depression, cardiac arrest, nephrotoxicity, hyperthermia, acute cerebral ischemia, and seizures [9, 10]. In addition, many cases of SCs-related deaths have been reported in many countries due to SCs toxicity, cardiovascular diseases, restrain asphyxia as a result of agitated delirium, organs failure, violent suicide, and traumatic accidents [11–13].

SCs are usually purchased in appealing packages containing one or more types of SCs and are marketed under names such as “Spice”, “K2”, “Nuclear Bomb”, “Scooby Snax”, “Mind Trip”, “Barely legal”, and “Green Giant” among others [14]. SCs are used recreationally and exist in the form of crystalline solids, which are normally white but can also be of other colours, or in a concentrated liquid form. The crystals are dissolved in ethanol or acetone and sprayed on natural herb mixtures or in cense in order to be smoked, and the concentrated liquid form can be ingested [15, 16]. More recently, SCs were found to be prepared in liquid-filled cartridges that can be used in electronic cigarettes (e-cigarettes) [17]. The herb mixtures used for smoking usually have no psychoactive effects. These include Damiana (*Turneradiffusa*) and herbs of the family Lamiaceae, such as *Melissa*, *Mentha*, and *Thymus* [18]. These preparations contain not only SCs, but many other compounds as well, including preservatives, additives, flavours, amides of fatty acids, and vitamin E [19]. In addition, SCs were also found to be mixed with other drugs including clenbuterol, *o*-desmethyltramadol, and mitragynine and benzodiazepines such as phenazepam, methamphetamine, tramadol, heroin, cannabis, and ketamine [14, 20, 21].

**Figure 1:**
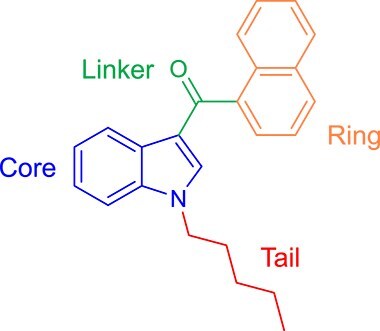
JWH-018 structure showing the four main structural components.

SCs are mostly lipophilic, nonpolar, and volatile when smoked as they are consisted of 22–26 carbon atoms with a side chain of 4–9 saturated carbon atoms [17]. The chemical structure of SCs is commonly composed of four main structural components (Figure 1) including the core (typically indoles or azaindoles such as indazole, benzimidazole and pyrrolopyridine), the linker (ketones, amides, and esters), the tail (alkyl, alicyclic, heterocyclic, aromatic, and heteroaromatic), and the ring (head group), which is the most diverse structure of the four components [1, 2, 17].

SCs have been classified by the UNODC according to their chemical structures into classical cannabinoids, non-classical cannabinoids, hybrid cannabinoids, aminoalkylindoles, naphthoylpyrroles, naphthylmethylindenes, and eicosanoids [22–25].

Classical cannabinoids are analogues of delta-9-tetrahydrocannabinol (∆^9^-THC), whereas non-classical cannabinoids are structurally dissimilar to ∆^9^-THC. However, hybrid cannabinoids have structural similarities to both classical and non-classical cannabinoids [24]. In order to subvert legal regulations, members of these classes are continuously modified, mostly by making small changes in the chemical structures such as changing an indole to indazole structure, e.g. AM-2201 to THJ-2201, or replacing the terminal fluorine [15]. Such structural changes have led to the production of the first (JWH-018), second (AM-2201, UR-1448), and third generation of SCs.

**Figure 2:**
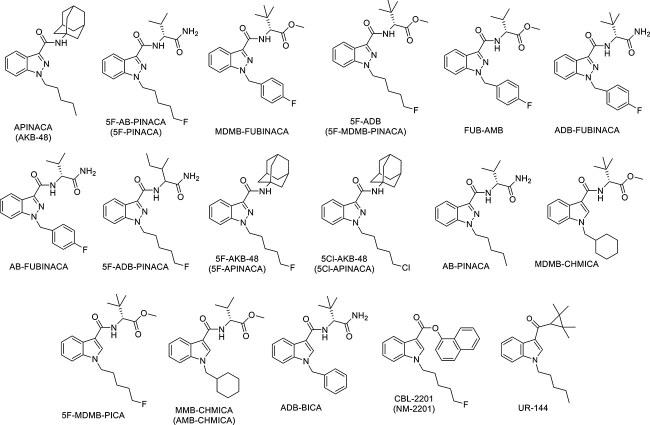
Indazole-3-carboxamide and indole-3-carboxamide families-related members detected previously [14].

Our previous study [14] has additionally reported the identification of more NPSs in Kuwait in 2018, including SCs and synthetic cathinones, using the gas chromatography–mass spectrometry (GC–MS) and the liquid chromatography-tandem mass spectrometry (LC–MS/MS) techniques. Detected SCs (Figure 2) include members of the indazole-3-carboxamide family such as APINACA (AKB-48), 5F-AB-PINACA, MDMB-FUBINACA, 5F-ADB, FUB-AMB, ADB-FUBINACA, AB-FUBINACA, 5F-ADB-PINACA, 5F-AKB-48, 5Cl-AKB-48, and AB-PINACA, whereas the identified substances MDMB-CHMICA, 5F-MDMB-PICA, MMB-CHMICA, and ADB-BICA are members of the indole-3-carboxamide family. Additionally, members of other families were identified, including CBL-2201, which belongs to the indole-3-carboxylate family, and UR-144, which belongs to the cyclopropylindole family.

However, these results are only tentative, which is one of the main limitations of using the GC–MS or the LC–MS/MS. Thus, in order to fully identify the compounds under investigation, the ideal techniques would be using nuclear magnetic resonance (NMR) or X-ray crystallography. Moreover, there are many other challenges in using the GC–MS or the LC–MS/MS, more specifically their limitations in unambiguously identifying different new SCs. These challenges have been addressed thoroughly in previous studies and will be highlighted here as the main purpose of our study is to overcome them.

## Materials and methods

### General experimental procedures

In our previous publications, we have discussed thoroughly the protocols and procedures that we are following in the Narcotics and Psychotropics Laboratory (NPL) in order to receive, analyse, and process different samples [14, 26]. Briefly, samples are received from different institutions in Kuwait in stamped and sealed envelopes in order to be examined and analysed by different instruments in laboratory for the detection of illicit drugs.

Routinely, all received samples are analysed by GC–MS as a confirmatory procedure. However, in some cases, more sophisticated instruments are required to ensure the accuracy of the results. Since some of these instruments are not available in our laboratory, we tend to get some assistance from other laboratories. Thus, NMR analyses and data interpretation were acquired through the Department of Pharmaceutical Chemistry, College of Pharmacy, Health Sciences Center, Kuwait University.

### Standard preparation

For GC–MS analyses, stock solutions containing all target compounds were prepared by pipetting 5 mL of each reference standard (100 mg/mL) into a 10-mL volumetric flask, and then diluting them with methanol to a final concentration of 50 mg/mL for each standard.

### Sample preparation

Approximately 500 mg of the powder or the plant materials in the seized samples were dissolved in 1 mL of methanol in a glass tube and centrifuged for 10 min at 1 253×g in a Hettich Universal 320 centrifuge (Andreas Hettich GmbH & Co. KG, Tuttlingen, Germany) at 21°C. Afterward, 250 mL of the supernatant were transferred to GC-vials for GC–MS analysis.

### GC–MS analyses

#### Chemicals and reagents

Methanol (MeOH; HiPerSolv CHROMANORM, HPLC grade, BDH prolabo) was purchased from VWR International (Fonenay-sous-Bois, France) and was used as the solvent for GC–MS analysis, as the blank control, for prewashing, for washing between the samples, and for post-sample injection washes. GC-vials, GC-vial lids, and GC-vial inserts were purchased from Agilent Technologies (Santa Clara, CA, USA). Standards for SCs were purchased from Cayman Chemicals (Ann Arbor, MI, USA), Chiron AS (Trondheim, Norway), and Lipomed (Arlesheim, Switzerland).

### Settings and protocol

GC–MS vials were placed into a GC 7693 (Agilent Technologies) with an autosampler and mass spectroscopy was performed with a 5977B GC/MSD (Agilent Technologies). The GC–MS parameters were determined using the methodology from our previous publications [14, 26]. The injection port temperature was set to 250°C, the splitless injection volume was 0.2 mL under a purge flow of helium gas at 3 mL/min, and the solvent delay was set to 3 min. The wash steps included four pre-injection washes, four post-injection washes, two sample washes, and six sample pumps. The initial temperature was set to 100°C for 4 min. Ramp 1 was set to 10°C/min until it reached 280°C, and was maintained for 2 min. The °C/min rate for Ramp 2 was set to 6°C until it reached 300°C, where it was maintained for 5 min. An HP-5MS UI column of 30 m length, 0.25 mm inner diameter, and 0.25 mm film thickness (Agilent, Waldbronn, Germany) was used with the flow rate set to 1 mL/min. The MS ionization mode was electron ionization (EI) set at 70 eV, with an ion source temperature of 280°C and an interface temperature of 290°C. Ions were monitored using SCAN mode. Cayman Spectral, Designer Drug Library, FORCHEM, and NIST 14 libraries were used.

### Chromatographic analyses

Thin layer chromatography (TLC) technique was used to analyse the seized samples. This analysis was done using pre-coated silica gel glass plates (5 × 10 cm, 250 m) with UV_254_ indicator and run in 3% MeOH in CHCl_3_. Visualization was performed *via* exposing the plates to the UV light (short wavelength; λ_max_ = 254 nm, CAMAG), followed by spraying them by *p*-anisaldehyde/sulfuric acid spray reagent and heating for 10 min.

### NMR analyses

The ^1^H and ^13^C NMR spectra were obtained on a Bruker Avance II-600 spectrometer (Bruker Corporation, Billerica, MA, USA) operating at 600 and 150 MHz, respectively. Both ^1^H and ^13^C NMR spectra were recorded in CDCl_3_ or CD_3_OD, and the chemical shift values were expressed in δ (ppm) relative to the internal standard TMS. For the ^13^C NMR spectra, spectral editing was determined by DEPT. 2D NMR data were obtained using the standard pulse sequence of the Bruker Avance II-600 for COSY, HSQC, and HMBC. Decoupled ^13^C NMR spectra were crucial in confirming the identity of some seized compounds as fluorinated ones through the calculations of ^1,2,3,4^*J*_C-F_ values.

## Results and discussion

**Figure 3:**
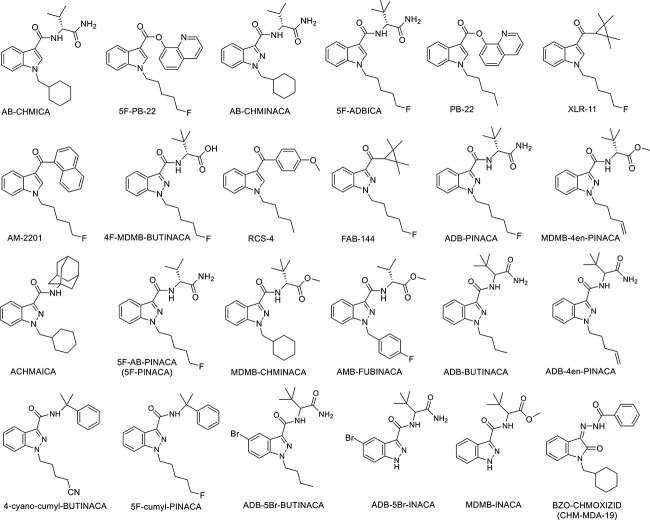
Synthetic cannabinoids recently seized and identified using GC–MS technique in Kuwait.

In order to identify other SCs members that are seized in Kuwait since our previous publication (17 members, see Figure 2), we analysed the data obtained from cases received at our laboratory from 2019 to 2023. Using the GC–MS, we identified 22 SCs members, which were not identified in our previous paper before 2019 [14] (Figure 3). This is in addition to the previously identified and reported 5F-PINACA and FUB-AMB (AMB-FUBINACA), including 13 members of the indazole-3-carboxamide family (ADB-5Br-BUTINACA, ADB-5Br-INACA, MDMB-INACA, ADB-BUTINACA, ADB-4en-PINACA, 4-cyano-cumyl-BUTINACA, 5F-cumyl-PINACA, ADB-PINACA, MDMB-4en-PINACA, ACHMINACA, MDMB-CHMINACA, 4F-MDMB-BUTINACA, AB-CHMINACA), 2 members of the indole-3-carboxamide family (AB-CHMICA and 5F-ADBICA), and 2 members of indole-3-carboxylate family (PB-22 and 5F-PB-22). Moreover, XLR-11 and FUB-144, which are cyclopropyl indoles, were also identified by NPL during 2019 until 2023, along with a member of naphthoyl indole family (AM-2201), benzoyl indole family (RCS-4), and oxindole with hydrazone linker, which was BZO-CHMOXIZID (CHM-MDA-19). This is in addition to the previously detected and reported 5F-PINACA and AMB-FUBINACA, known also as FUB-AMB, as shown in Figure 2.

**Figure 4:**
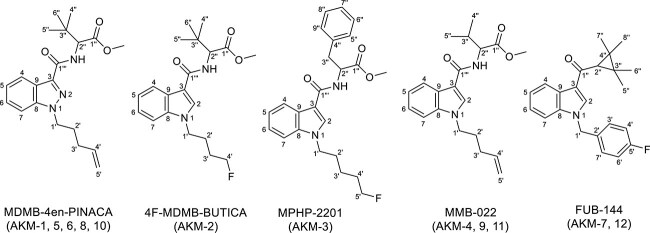
Synthetic cannabinoids recently seized and identified using 1D- and 2D-NMR in Kuwait.

Seventeen seized SC samples were sent to the Department of Pharmaceutical Chemistry, College of Pharmacy, Kuwait University, for spectral analyses and identification. The samples were tested for their purity using TLC. Twelve samples, abbreviated from AKM-1 to AKM-12, were found to be pure enough to run NMR analyses. Different NMR spectra were acquired including ^1^H, ^13^C, DEPT 45°, 90°, 135°, COSY, HSQC, and HMBC. The identities of five samples (AKM-1, 5, 6, 8, 10) were confirmed as MDMB-4en-PINACA, one sample (AKM-2) as 4F-MDMB-BUTICA, one sample (AKM-3) as MPHP-2201, three samples (AKM-4, 9, 11) as MMB-022, and two samples (AKM-7, 12) as FUB-144 (Figure 4).

Seventeen samples, potentially SCs, were received for analysis and possible detection of illicit drugs. The previously published standard protocol was followed [14, 26]. Samples were prepared and analysed by GC–MS, and the results were compared with those in Cayman Spectral Library, Designer Drug Library, FORCHEM, and NIST 14 library. This comparison tentatively revealed the identity of samples AKM-1, 5, 6, 8, 10 and 11 as the known compound MDMB-4en-PINACA, AKM-2 as the known 5F-MDMB-PICA, AKM-3 as the known MMB-2201, and AKM-4 and 12 as the known MMB-022. While samples AKM-7 and 9 were identified as the known compound FUB-144. Other samples were impure or a mixture of several SCs.

After these 12 samples (AKM-1–AKM-12) were confirmed to be pure, we proceeded with 1D- and 2D-NMR analyses.

Samples AKM-1, 5, 6, 8, and 10: chromatographic analyses and NMR spectra showed that samples AKM-1, 5, 6, 8 and 10 are identical and have a molecular formula of C_20_H_27_N_3_O_3_. ^13^C NMR spectra showed that these samples are identical and belong to the indazole-3-caroxamide family of SCs. Seventeen resonances were shown in the ^13^C NMR spectra, with the possibility of the presence of three identical carbons (δ_C_ 26.9). These resonances were assigned to C-4^′^, 5^′^^′^, and 6^′^^′^. Two resonances were in the carbonyl groups region (C-1^′″^ at δ_C_ 162.5 and C-1^′^^′^ at δ_C_ 172.4). The carbon resonances of the indazole system, along with the terminal ethenyl moiety, were in the aromatic/olefinic region (δ_C_ 109.4–141.1 ppm). The other aliphatic carbons resonated in the aliphatic and oxygenated aliphatic regions (δ_C_ 26.9–59.7 ppm). Resonance at δ_C_ 26.9 ppm was with a high intensity and showed a cross-peak in HSQC spectrum with a sharp singlet proton resonated at δ_H_ 1.11 and integrated for 9 Hs. This confirmed the assignments of these three methyl groups (C-4^′^^′^, 5^′^^′^, and 6^′^^′^). All other ^1^H and ^13^C resonances were unambiguously assigned using HSQC and HMBC spectra. This revealed the identity of compounds AKM-1, 5, 6, 8, and 10 to be MDMB-4en-PINACA. Its chemical name is methyl (*S*)-3,3-dimethyl-2-(1-(pent-4-en-1-yl)-1*H*-indazole-3-carboxamido)butanoate (Figure 4). The NMR data (Table 1) were indistinguishable from the reported ones [27]. The identities of these samples were consistent with those acquired from GC–MS analysis.

**Table 1:** ^1^H and ^13^C NMR data (600 MHz, CDCl_3_) for compounds AKM-1, 2, 3, 4, and 7.

No	AKM-1^a^		AKM-2		AKM-3		AKM-4		AKM-7	
	^13^C, m^a^	^1^H	^13^C, m	^1^H	^13^C, m	^1^H	^13^C, m	^1^H	^13^C, m	^1^H
2	-	-	132.1, d	7.77, s	132.2, d	7.76, s	132.0, d	7.77, s	133.8, d	7.69, s
3	136.9, s	-	110.7, s	-	110.3, s	-	110.7, s	-	120.5, s	-
4	122.8, d	7.57, d (8.6)	120.1, d	7.97, dd (4.8, 1.8)	120.4, d	7.77, d (8.4)	120.3, d	8.02, dd (4.2, 1.8)	122.6, d	8.45, dd (5.4, 1.8)
5	123.0, d	7.27, m	121.9, d	7.27, m	121.8, d	7.22, m	121.8, d	7.29, m	123.0, d	7.28, m
6	126.9, d	7.41, dd (7.8)	122.8, d	7.31, m	122.8, d	7.27, m	122.7, d	7.31, m	123.5, d	7.25, m
7	109.4, d	8.36, dd (8.4, 1.2)	110.5, d	7.41, m	110.4, d	7.38, d (8.4)	110.5, d	7.39, d (8.4)	110.1, d	7.26, dd (5.4, 1.2)
8	141.1, s	-	136.7, s	-	136.3, s	-	136.8, s	-	136.9, s	-
9	123.1, s	-	125.4, s	-	125.5, s	-	125.6, s	-	126.7, s	-
										
1′	48.8, t	4.42, dd (7.2, 6.9)	46.5, t	4.19, t (6.6)	46.9, t	4.16, 2H, t (7.2)	46.2, t	4.16, dd (7.2, 7.2)	50.2, t	5.34, s
2′	28.9, t	2.09,m	26.4, t (^3^*J*_C-F_ = 3 Hz)	2.02, m	29.8, t	1.92, 2H, m	30.9, t	1.99, m	132.1, s (^4^*J*_C-F_ = 3 Hz)	-
3′	30.9, t	2.13, m	27.8, t (^2^*J*_C-F_ = 21 Hz)	1.69, m	23.0, t (^3^*J*_C-F_ = 4.5 Hz)	1.48, 2H, m	31.9, t	2.11, m	128.8^a^, d (^3^*J*_C-F_ = 9 Hz)	7.15^b^, dd (5.4, 4.8)
4′	137.2, d	5.82, m	83.6, t (^1^*J*_C-F_ = 164 Hz)	a: 4.48, d (6.0) b: 4.41, d (6.6)	30.1, t (^2^*J*_C-F_ = 19.5 Hz)	1.73, 2H, m	137.1, d	5.81, m	116.2^b^, d (^2^*J*_C-F_ = 22.5 Hz)	7.04^c^, dd (9.0, 9.0)
5′	116.1, t	a: 5.08, dd (13.5, 1.8) b: 5.05, dd (5.4, 1.8)	-	-	83.8, t (^1^*J*_C-F_ = 164.5 Hz)	a: 4.39, dd (7.2, 7.2) b: 4.47, dd (7.2, 7.2)	116.3, t	a: 5.08, dd (13.4, 1.8) b: 5.05, dd (5.4, 1.8)	162.7, s (^2^*J*_C-F_ = 22.5 Hz)	-
6′	-	-	-	-	-	-	-	-	116.2^b^, d (^1^*J*_C-F_ = 246 Hz)	7.04^c^, dd (9.0, 9.0)
7′	-	-	-	-	-	-	-	-	128.8^a^, d (^3^*J*_C-F_ = 9 Hz)	7.15^b^, dd (5.4, 4.8)
										
1″	172.4, s	-	172.8, s	-	172.7, s	-	173.4, s	-	194.9, s	-
2″	59.7, d	4.75, d (9.6)	60.0, d	4.76, d (9.0)	53.4, d	5.19, br d (3.0)	57.1, d	4.88, dd (8.4, 4.8)	42.0, d	1.95, s
3″	35.2, s	-	35.2, s	-	38.3, t	a: 3.28, dd (9.0, 4.8) b: 3.35, dd (9.0, 4.8)	29.1, d	2.32, m	32.1^b^, s	-
4″	26.9^b^, q	1.11^b^, s	26.9^b^, q	1.10^b^, s	136.7, s	-	18.3, q	1.04^b^, d (7.2)	32.1^b^, s	-
5″	26.9^b^, q	1.11^b^, s	26.9^b^, q	1.10^b^, s	128.8^b^, d	7.32^b^, m	19.3, q	1.07^b^, d (7.2)	24.2^c^, q	1.30^d^, s
6″	26.9^b^, q	1.11^b^, s	26.9^b^, q	1.10^b^, s	129.5^c^, d	7.21^c^, m	-	-	24.2^c^, q	1.30^d^, s
7″	-	-	-	-	127.3, d	7.28, m	-	-	17.2^d^, q	1.37^e^, s
8″	-	-	-	-	129.5^c^, d	7.21^c^, m	-	-	17.2^d^, q	1.37^e^, s
9″	-	-	-	-	128.8^b^, d	7.32^b^, m	-	-	-	-
										
1″′	162.5, s	-	165.1, s	-	164.8, s	-	165.2, s	-	-	-
OCH_3_	52.0, q	3.77, s	52.0, q	3.76, s	52.6, q	3.79, s	52.4, q	3.80, s	-	-
NH	-	7.57, d (9.6)	-	7.36, d (8.4)	-	6.5, br s	-	6.53, d (9.0)	-	-

a Carbon multiplicities were determined by DEPT experiments. ^b,c,d,e^Assignments within the same column are interchangeable. s: singlet; d: doublet; t: triplet; dd: double doublet; q: quartet; m: multiplet; br: broad.

Sample AKM-2: on the other hand, ^13^C NMR spectra showed that this compound is an indole-3-carboxamide type of the SCs with a molecular formula of C_20_H_27_FN_2_O_3_. The NMR data showed that its head part (C-1^′^^′^–C-6^′^^′^) is identical to that of AKM-1. Moreover, ^13^C NMR spectra indicted that this compound is a C-4^′^ fourinated derivative due to the presence of ^1,2,3^*J*_C-F_ couplings (Table 1). Other ^1^H and ^13^C NMR data assigments were aided by HSQC, HMBC and COSY experiments. This confirmed the identity of AKM-2 to be 4F-MDMB-BUTICA which has the chemical name methyl (*S*)-2-(1-(4-fluorobutyl)-1*H*-indole-3-carboxamido)-3,3-dimethylbutanoate (Figure 4) [28]. The NMR analyses established that the identity of this sample differs from the one obtained from GC–MS analysis, where the sample was tentatively identified as 5F-MDMB-PICA.

Sample AKM-3: likewise, this compound was shown to be a fourinated indole-3-carboxamide derivative with a moleuclar formula of C_24_H_27_FN_2_O_3_. NMR data comparison with those of AKM-2 revealed that both compounds have identical linker, core and almost the same tail. The tail of AKM-3 has one extra carbon. AKM-3 showed a different head with that of AKM-2. ^13^C NMR spectra showed 22 different resonances with the possibility of having two sets of two identical carbons with almost double intensities. Fourteen of these resonaces were in the aromatic region, indicating the possibility of having an aromatic ring in the head part. Additionally, C-3^′^, 4^′^, and 5^′^ of the tail resonated in the ^1^H-decoupled ^13^C NMR spectra as doublets with ^3^*J*_C-F_, ^2^*J*_C-F_, and ^1^*J*_C-F_ couplings, respectively (Table 1). All protons and carbons were assigned unambiguously. AKM-3 identity was revealed to be the known MPHP-2201. Its chemical name is methyl (*S*)-2-(1-(5-fluoropentyl)-1*H*-indole-3-carboxamido)-3-phenylpropanoate [28]. Again, the NMR analyses established that the identity of this sample differs from the one obtained from GC–MS analysis, where the sample was tentatively identified as MMB-2201.

Samples AKM-4, 9, and 11: thin layer chromatography and NMR analyses revealed that samples AKM-4, 9, and 11 are identical and were very close to AKM-1 in the head, linker, and tail moieties. The NMR data showed that these samples belong to the indole-3-carboxamide family of the SCs with a molecular formula of C_20_H_26_N_2_O_3_. The tail was shown to have a terminal alkene (C4^′^ resonating at δ_C_ 137.1 as a doublet and C-5^′^ at δ_C_ 116.3 as a triplet). Moreover, the head was shown to have an isopropyl moiety as indicated from the presence of two doublets in the ^1^H NMR spectra each integrated for 3 Hs and appeared at δ_H_ 1.04 and 1.07 ppm (H-4^′^^′^ and 5^′^^′^). In the COSY spectrum, these two methyl groups showed a cross-peak correlation with a single proton resonated as multiplet at δ_H_ 2.32 ppm (H-3^′^^′^). All ^1^H and ^13^C NMR resonances were unambiguously assigned and revealed that these sample (AKM-4, 9, and 11) are the known MMB-022. Chemically, it is methyl(*S*)-2-(1-(pent-4-en-1-yl)-1*H*-indole-3-carboxamido)-3-methylbutanoate [28]. The NMR analyses established that the identities of these samples differ from the ones obtained from GC–MS analysis, where samples AKM-9 and 11 were tentatively identified as FUB-144 and MDMB-4en-PINACA, respectively.

Samples AKM-7 and 12: samples AKM-7 and 12 were shown to be identical to each other and belong to the indole-3-ketone family of the SCs with a molecular formula of C_23_H_24_FNO. The presence of a ketone moiety rather than a carboxamide one was clear from the resonance of C-1^′^^′^ (C=O), as a singlet, at a more de-shielded position (δ_C_ 194.9 ppm) than the regular amide carbonyl (at δ_C_ 173.0) as a singlet. In addition, C-3 resonated, as a singlet, at a more deshielded position (δ_C_ 120.5 ppm) in comparison to δ_C_ 110.0 ppm seen in the previous carboxamide-containing compounds. Moreover, these samples were shown to possess the unique fully methylated cyclopropyl system as the head, and a fluorinated benzyl group as the tail. The presence of a fluorinated benzyl derivative can be concluded from the resonance pattern of all the phenyl carbons, which appeared each as a doublet with ^4^*J*_C-F_, ^3^*J*_C-F_, ^2^*J*_C-F_, and ^1^*J*_C-F_ couplings, as seen in Table 1. All other ^1^H and ^13^C resonances were unambiguously assigned. The identity of these samples were confirmed to be the known FUB-144 [29], and were consistent with those acquired from GC–MS analysis. It is chemically known as (1-(4-fluorobenzyl)-1*H*-indol-3-yl)-(2,2,3,3-tetramethylcyclopropyl)methanone.
